# Same-day discharge versus overnight stay after catheter ablation for atrial fibrillation: a systematic review and meta-analysis

**DOI:** 10.1007/s10840-026-02286-x

**Published:** 2026-05-18

**Authors:** Martín Rebolledo Del Toro, Maria Alejandra Robledo Gomez, Oscar Mauricio Muñoz-Velandia, Juan Felipe Betancourt Rodriguez

**Affiliations:** 1 Division of Electrophysiology, Hospital Universitario San Ignacio, Bogota, Colombia; 2 Department of Internal Medicine, Pontificia Universidad Javeriana, Bogota, Colombia; 3 Department of Internal Medicine, Hospital Universitario San Ignacio, Bogota, Colombia

**Keywords:** Atrial fibrillation, Catheter ablation, Same day discharge, Ambulatory care, Systematic review, Meta-analysis

## Abstract

**Background:**

The safety of same day discharge after atrial fibrillation ablation remains uncertain because prior evidence relied mainly on heterogeneous observational studies with limited control of confounding. This systematic review and meta-analysis compared structured same day discharge with overnight stay.

**Methods:**

We searched MEDLINE, Embase, Web of Science, Scopus, CENTRAL, Epistemonikos and LILACS from 1994 to May 2025 for randomized and nonrandomized controlled studies of adults undergoing atrial fibrillation ablation with pulmonary vein isolation. Comparisons were protocolized same day discharge versus overnight hospitalization. Outcomes were all-cause mortality, short term (30-day) unplanned medical contact (readmissions or emergency visits), and procedural complications. Random-effects models generated pooled odds ratios (ORs) with 95% confidence intervals.

**Results:**

Nineteen studies (26,335 patients) met inclusion criteria, including two randomized trials and seventeen non-randomized studies. Same day discharge success averaged 89% across structured programs. Mortality was rare in both groups, and pooled mortality did not differ between strategies. At 30 days, same day discharge was not associated with excess of readmission or emergency department visits and the pooled estimate favored same day discharge (OR 0.63; 95% CI 0.51–0.78; I^2^ 0%). Complication rates were low in both groups, although definitions varied. Most non-randomized studies had moderate to serious risk of bias.

**Conclusion:**

Structured same day discharge after atrial fibrillation ablation shows no evidence of increased mortality, major complications, or unplanned healthcare use in selected outpatients. Implementation should rely on explicit eligibility criteria and early follow up. Further randomized trials are required to establish safety in higher-risk populations and to evaluate the safety of newer ablation technologies.

**Supplementary Information:**

The online version contains supplementary material available at https://doi.org/10.1007/s10840-026-02286-x.

## Introduction

Atrial fibrillation (AF) is a progressive arrhythmia associated with substantial morbidity and rising global prevalence [1]. Its clinical impact is driven by risks of stroke, heart-failure decompensation, and impaired quality of life, situating rhythm control as a central therapeutic objective [2–4]. Catheter ablation, primarily through pulmonary vein isolation (PVI), has become an established rhythm‑control strategy and is recommended by contemporary guidelines as an effective option for selected patients [2–6].

The increasing volume of procedures and the ongoing pressure on hospital capacity have accelerated interest in ambulatory pathways after AF ablation, including same-day discharge (SDD) [6]. The rationale for SDD is based on the temporal pattern of post-procedural risk, with most major complications occurring within the first few hours, and being rare thereafter once haemostasis, clinical stability and ambulation criteria have been met [7] Additionally, advances in periprocedural management and vascular‑access techniques have supported early mobilization without compromising safety [6], and observational cohorts from various centers have demonstrated feasibility and shown consistency in the outcomes obtained in the initial follow-up phases after all ablation modalities [8–11].

Three systematic reviews published in 2022 reported no significant differences in short-term complications or readmissions between SDD and overnight stay (ONS) [12–14]. However, all relied exclusively on retrospective cohorts, used discharge timing rather than protocolized SDD as the exposure, and lacked adequate control of confounding, limiting confidence in their conclusions. More recent prospective studies and at least two randomized trials have expanded the evidence base, reporting favorable safety signals while highlighting the need for updated synthesis in light of evolving practice [15–19].

Despite accumulating data, SDD adoption remains inconsistent. European surveys report limited routine implementation, whereas a recent North American survey suggests broader uptake with ongoing concerns about suitability in ambulatory surgical centers [6, 20–22]. Unresolved questions include the comparative safety of SDD versus ONS across patient profiles, procedural approaches, and institutional protocols [12–14]. To address these unresolved questions and incorporate the most recent prospective cohorts and randomized trials published after prior reviews, we conducted a systematic review and meta-analysis evaluating the safety and effectiveness of protocolized SDD compared with overnight stay after AF ablation.

## Materials and methods

### Protocol and registration

The protocol was registered in the International Prospective Register of Systematic Reviews (PROSPERO; CRD42024572030; July 22, 2024). This systematic review and meta-analysis followed Cochrane methodology for reviews of interventions [23], and is reported according to the Preferred Reporting Items for Systematic Reviews and Meta-Analyses (PRISMA 2020) and the Meta-analysis Of Observational Studies in Epidemiology (MOOSE) recommendations [24, 25]. The protocol was approved by our institutional Research Ethics committee (Act number 17/2024, document FM-CIE-1090-24).

## Eligibility criteria

We included randomized controlled trials (RCTs) and nonrandomized studies comparing management protocols including same-day discharge (SDD) with overnight stay (ONS) in adults (≥ 18 years) with atrial fibrillation (AF) undergoing catheter ablation with pulmonary vein isolation using any energy source (radiofrequency, cryoablation, or pulsed-field ablation). Studies had to describe the SDD protocol workflow and discharge criteria in sufficient detail and compare SDD with standard care involving at least one overnight hospital stay. Eligible studies had to report at least one safety outcome, including all-cause mortality, unplanned medical contact (hospital readmission or emergency department visit), or procedure-related complications. No language restrictions were applied. We excluded reviews, conference abstracts, editorials, case reports or series of cases, before–after studies, single-arm cohorts, hybrid surgical–catheter procedures, studies with unclear outcome definitions, and studies not reporting AF ablation outcomes separately. Studies reporting only intraprocedural outcomes without post-discharge follow-up were excluded. We also excluded studies based solely on administrative or claims databases, in which exposure (SDD vs. ONS) and outcomes were defined only by billing codes and calendar dates without individual clinical adjudication and with limited information on patient selection, procedural characteristics, or discharge criteria, This is because those studies could introduce relevant exposure and outcome misclassification, as well as substantial residual confounding, which would compromise the internal validity of comparative safety and effectiveness analyses. Finally, studies that classified SDD only based on observed discharge timing, without a prespecified SDD protocol with explicit eligibility criteria and workflow, were excluded because in such designs SDD represents a discharge status rather than a reproducible intervention and is highly susceptible to selection and indication bias.

## Information sources and search strategy

We performed a comprehensive literature search in PubMed (MEDLINE), Embase (Elsevier), Web of Science, Scopus, Cochrane CENTRAL, Epistemonikos, and BVSalud (LILACS). The initial search was conducted on July 30, 2024, and updated on May 24, 2025. The strategy combined controlled vocabulary (MeSH/Emtree) and free-text terms for “atrial fibrillation,” “catheter ablation,” “pulmonary vein isolation,” and “same-day discharge,” using Boolean operators (AND/OR). Validated text filters were applied to identify randomized and nonrandomized controlled studies, and the search was limited to adult populations (≥ 19 years) [26–28]. The time window started in 1994, because catheter ablation for AF was first described in the mid-1990s and no earlier studies were expected [29, 30]. Reference lists of all included articles were screened to identify additional eligible studies [23]. Full research strategy and terms is described in our supplementary appendix.

## Study selection and data collection

Records were deduplicated in EndNote and screened in Rayyan [31, 32]. Two reviewers (MRdT and MARG) independently screened titles/abstracts and full texts; disagreements were resolved by consensus or with the help of a third reviewer (OMM). Data were extracted in duplicate into a standardized Microsoft Excel form capturing study design, setting, population, demographic and baseline clinical characteristics, procedural details, SDD protocol components, follow-up duration, and outcomes. Outcomes included all-cause mortality, unplanned medical contact, defined as hospital readmission or emergency department visit, and complications or adverse events. For unplanned medical contact, 30-day follow-up was prioritized as the primary window; different time horizons were treated as exploratory. When reported, we recorded SDD success rate, defined as the proportion of patients discharged on the day of ablation among those planned for SDD according to the study protocol. When both adjusted and unadjusted estimates were reported, adjusted estimates were preferentially extracted.

## Risk of bias assessment

Randomized trials were assessed with the Cochrane Risk of Bias 2 tool (RoB 2, 2019) [33]. Nonrandomized studies were assessed with the ROBINS-I V2 tool (2024 update) [34, 35]. Two reviewers (MRdT and MARG) performed independent assessments, which were verified by a third reviewer (OMM). Risk-of-bias visualizations were generated in RevMan Web and robvis [36, 37].

### Data synthesis and statistical analysis

Dichotomous outcomes were pooled as odds ratios (ORs) with 95% confidence intervals (CIs). Random-effects meta-analyses with inverse-variance weighting were performed in RevMan Web [36]. Heterogeneity was quantified with Cochran’s Q and the I² statistic; I² > 50% was considered substantial. Subgroup analyses were prespecified according to follow-up time, study design (RCT vs. non-RCT), AF type (paroxysmal vs. persistent), ablation energy (radiofrequency, cryoablation, pulsed-field), and key SDD protocol features (timing of SDD assignment, monitoring duration, imaging prior to discharge, and hemostasis strategy). Analyses by AF type, monitoring duration, and hemostasis strategy were prespecified, but none of the included studies reported outcomes stratified according to these protocol features, precluding these subgroup analyses. Similarly, only a few studies used a single ablation energy source and reported the outcomes of interest, so exploratory subgroup meta-analyses by radiofrequency versus cryoballoon ablation were underpowered and not pursued. An exploratory study-level subgroup analysis comparing protocols that mandated routine pre-discharge transthoracic echocardiography with those that did not, is presented. The evaluation of publication bias using funnel plots and Egger’s test was planned, however it was not performed as ≤ 10 studies were available per outcome within each time window, according with Cochrane recommendations [23]. Adjusted effect estimates were preferentially extracted when reported; otherwise, crude effect sizes were calculated from events and totals. Studies with zero events in both arms were excluded from pooled effect estimation but retained for descriptive summaries.

## Results

### Study selection and baseline characteristics

**Fig. 1:**
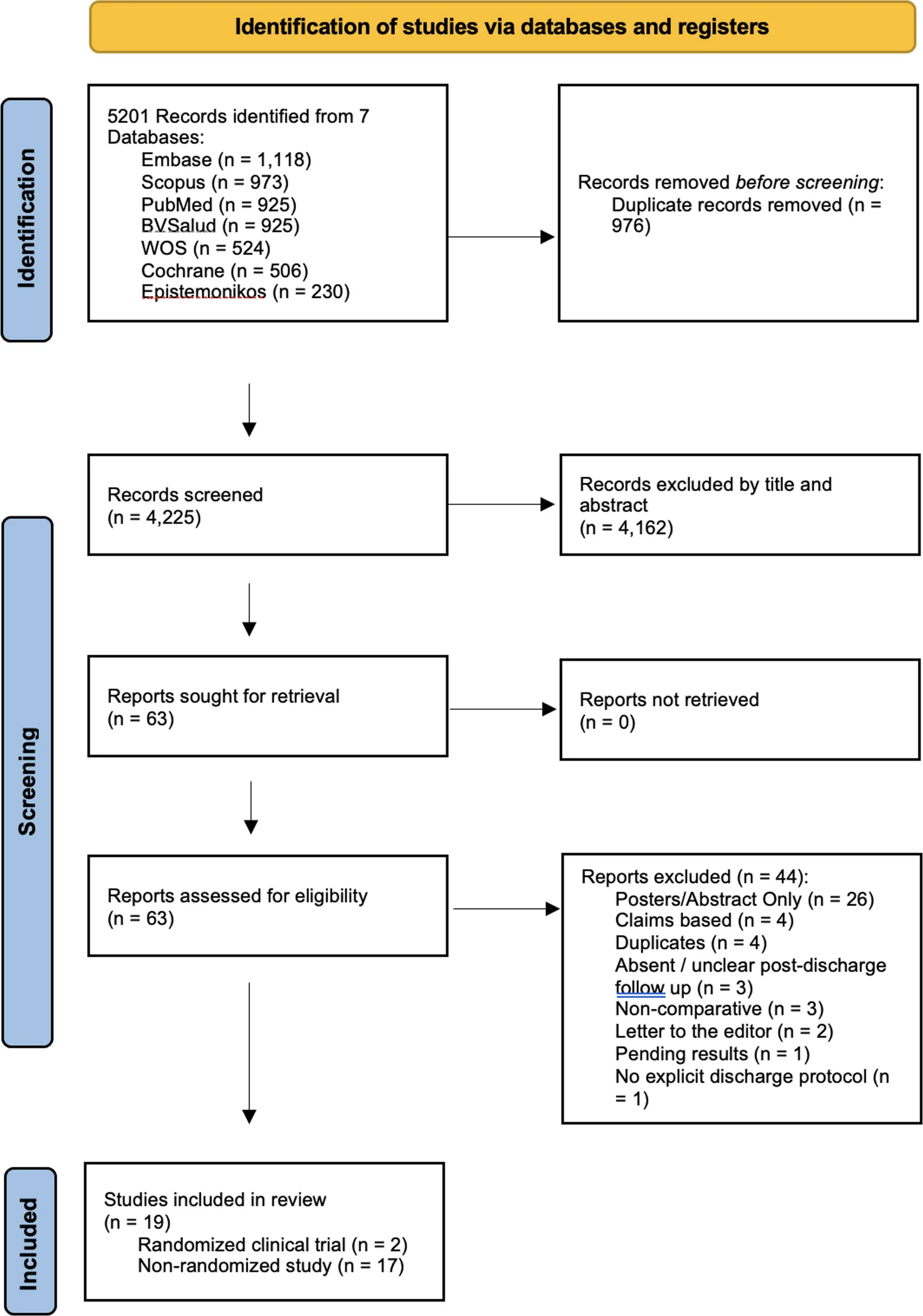
PRISMA flow diagram

We identified 5,201 records, removed 976 duplicates, screened 4,225 titles and abstracts, and assessed 63 full-text articles. Finally, nineteen studies met inclusion criteria, including two randomized controlled trials [17, 18] and 17 controlled non-randomized studies [8–11, 15, 19, 38–48] (Fig. 1). Included studies were published between 2010 and 2025 and enrolled patients from North America, Europe, Asia-Pacific, and South America. The combined sample comprised 26,335 patients.

Baseline characteristics are summarized in Table 1. Most cohorts included patients with paroxysmal or persistent AF undergoing left atrial ablation, often incorporating both index and redo procedures. Men represented approximately two thirds of the population. The weighted mean age across studies reporting was 62.2 years. Heart failure, reduced left ventricular ejection fraction, higher CHA₂DS₂-VASc scores, and longer procedure times tended to be more frequent among patients who remained overnight.

**Table 1:** Description of the studies

Study, year,	Time and location	Design Type. Follow up, d		Main Inclusion Criteria	Included Patients, *n* (Total & groups)	Age in years, Mean (SD)or Median (IQR)	Men, %		Parox AF, %		Previous ablation, %	Cardiomyopathy LVEF HF, % Mean (SD) or Median (IQR)		CHA_2_DS_2_VASc (%) or mean ± (SD)		Procedure time, min, Mean ± SD	
1. Mondragon et al. [38]	Argentina Jun 2015 – May 2016	Prosp SC	30	Parox/Pers AF refractory/intolerant AAD	T 195 SDD 58 ONS 137	SDD 57 (49–66) ONS 62 (52–66)		SDD 84.5 ONS 75.2		SDD 79.3 ONS 78	SDD 20 ONS 19.6	SDD 60 (59–64) ONS 60 (50–62)	NR		SDD 0 = 31 1 = 41.4 2 = 17.2 ≥ 3 = 10.3 ONS 0 = 31.4 1 = 27.7 2 = 24.1 ≥ 3 = 16.7	91.5*	
2. Opel et al. [39]	UK Jun 2014–2016	Prosp MC	90, 420	Sx Parox/Early Pers AF (< 1y) refractory ≥ 1 AAD	T 552 SDD 276 ONS 276	SDD 61 ± 0.7 ONS 60 ± 0.8		SDD 61 ONS 60		SDD 79 ONS 81	0 (excl)	NR	NR		SDD 0 = 26 1 = 20 2 = 26 ≥ 3 = 28 ONS 0 = 26 1 = 25 2 = 22 ≥ 3 = 27	SDD 63.5 ± 1.1 ONS 101.7 ± 2.9	
3. Bartoletti et al. [40]	UK Apr 2014 – Mar 2017	Retro SC	90	All AF abl (1st time or redo) w/LA access attempted	T 785 SDD 143 ONS 642	SDD 9 ± 11 ONS 59 ± 11		SDD 68.5 ONS 68.1		SDD 75 ONS NR	NR	NR	NR		SDD 2 ± 1 ONS 1 ± 1	SDD 120 ± 29 ONS 153 ± 43	
4. Akula et al. [41]	USA Jan 2005 – Dec 2015	Retro SC	90	AL AF or left AFL. Excl: inpatient and isolated right AFL	T 571 SDD 426 ONS 145	SDD 62.3 ONS 60.2		SDD 52 ONS 60		SDD 56.7 ONS 72.5	0 (excl)	NR	NR		NR	NR	
5. Reddy et al. [42]	UK Mar 2017 – Mar 2018	Retro SC	90, 180	Sx Parox/Pers (de-novo/redo)	T 448 SDD 168 ONS 452	SDD 60.1 ± 9.9 ONS 60.3 ± 10.0		SDD 70.8 ONS 72.9		SDD 78 ONS 64.4	SDD 22 ONS 28.6	NR	NR		NR	SDD 139.6 ± 37.8 ONS 160.7 ± 48.6	
6. Deyell et al. [43]	Canada Jan 2010 – Dec 2014	Retro MC	30, 90, 180, 360	Outpt AF abl (index & redo), ≥ 19 years	T 3,054 SDD 2,418 ONS 636	60.4 ± 9.5		72.8		62.4	39.2	NR	NR		NR	190 (65–483)*	
7. Creta et al. [11]	UK Jun 2016 – Nov 2019	Retro MC	2, 30, 90	All AF abl	T 2628 SDD 727 ONS 1901	SDD 61.1 ± 12.5 ONS 62.9 ± 11.3		SDD 69.6 ONS 60.1		SDD 58 ONS 48.8	SDD 13.8 ONS 40.4	NR	SDD 10.9 ONS 15.5		NR	SDD 79 ± 42 ONS 150 ± 83	
8. Kowalski et al. [10]	USA Jan 2014 – Mar 2019	Retro Match MC	30	All AF Cryo abl	T 2374 SDD 1194 ONS 1180	SDD 64 ± 11 ONS 66 ± 10		SDD 69 ONS 67		NR	NR	SDD 56 ± 9 ONS 55 ± 10	SDD 10 ONS 16		SDD 1.4 ± 1.0 ONS 2.2 ± 1.4	NR	
9. Rajendra et al. [43]	USA Jan 2017 – Jun 2018	Prosp Match SC	1, 7, 90	Parox AF, < 75y, BMI < 40, CHA_2_DS_2_VASc < 4	T 82 SDD 41 ONS 41	SDD 58.5 ± 9.9 ONS 59.9 ± 12.8		SDD 61 ONS 61		SDD 100 ONS 100	SDD 7.3 ONS 4.9	NR	SDD 4.9 ONS 0		SDD 0 = 19.5 1 = 26.8 2 = 34.1 ≥ 3 = 19.5 ONS 0 = 19.5 1 = 31.7 2 = 24.4 ≥ 3 = 24.4	SDD 59 ± 15 ONS 67 ± 22	
10. He et al. [8]	UK Jan 2011 – Dec 2019	Retro MC	1, 120	Complex LA abl (AF ± LA FL/AT)	T 967 SDD 328 ONS 639	SDD 62.5 ± 10.8 ONS 59.7 ± 12.1		SDD 45 ONS 43		SDD 65 ONS 64	SDD 7.7 ONS 15.0	NR	SDD 45 ONS 43		NR	SDD 146.2 ± 80.9 ONS 169.2 ± 71.8	
11. Brown et al. [45]	USA Aug 2017 – Aug 2019	Parall MC	3	LA alb (AF, FL, AT)	T 409 SDD 257 ONS 199	SDD 64.7 (11.1) ONS 63.1 (10.9)		SDD 68.1 ONS 67.3		SDD 61 ONS 48.7	SDD 34.4 ONS 31.2	SDD 54.5 ± 8.35 ONS 50.9 ± 11.12	SDD 14.8 ONS 26.6		NR	SDD 134 ± 36 ONS 129 ± 31	
12. Deyell et al. [15]	Canada Jan 2018 – Oct 2019	Retro MC	30, 90, 180, 360	≥ 19 y AF abl VCHR residents w/complete data	T 421 SDD 381 ONS 40	T 63.3 ± 10.2		T 67		T 56.5	T 26	NR	T 15.9		NR	RF 211 (87–404)* Cryo 138 (104–183)*	
13. Sangrigoli et al. [17]	USA Nov 2018 – Oct 2020	RCT MC	30	≥ 18y, parox AF Cryo abl.	T 45 SDD 22 ONS 23	SDD 58.5 ± 12.0 ONS 61.96 ± 7.84		SDD 68 ONS 74		SDD 100 ONS 100	NR	NR	NR		NR	SDD 85.6 ± 18.7 ONS 81.1 ± 19.6	
14. Eldadah et al. [46]	USA NR	Prosp MC	2, 4, 15 ± 5	≥ 18 Parox/persist AF Abl + VASCADE MVP VCD	T 354 SDD 323 ONS 31	SDD 64.5 ± 10.6 ONS 66.6 ± 11.1		SDD 71.5 ONS 71.0		T 75.3 SDD 42.7 ONS 41.9	T 31.1 SDD 32.2 ONS 19.4	NR	T 23.4 SDD 22.6 ONS 32.3		T 2.4 SDD 2.3 ONS 3.1	NR	
15. Asbeutah et al. [47]	USA Jan–Jun 2019 and Jan–Jun 2021	Retro MC	30	All AF abl pre-pandemic & pandemic window (minimum stay policy)	T 225 SDD 116 ONS 134	T 64.5 ± 9.0 y		T 70.2		T 57	NR	NR	NR		NR	NR	
16. Rajendra et al. [44]	USA Nov 2020 – Jun 2022	Prosp MC	1, 30, 90, 180. 360	Parox/persist AF RF abl SDD planned per protocol vs. non-SDD if ineligible	T 2,332 SDD 1,982 ONS 350	SDD 66.5 ± 10.5 ONS 68.0 ± 10.4		SDD 60.3 ONS 55.2		SDD 55 ONS NR	NR	SDD 54.7 ± 10.9 ONS 51.0 ± 13.2	T 19.6 SDD 17.8 ONS 30		T 2.8 ± 1.6 SDD 2.8 ± 1.6 ONS 3.1 ± 1.6	SDD 93 ± 45 ONS 113 ± 54	
17. Castro-Urda et al. [18]	Spain Jan 2021 – Jun 2022	RCT SC	7, 30	18–80 y, parox/persist outpt abl Proglide SDD vs. no VCD ONS	T 100 SDD 50 ONS 50	SDD 65 (58.8–69.8) ONS 63 (54.3–68)		SDD 64 ONS 74		T 57 SDD 58 ONS 56	T 8 SDD 8 ONS 8	SDD 56 (I55–60.25) ONS 57 (54–65.5)	T 23 SDD 22 ONS 26		NR	NR	
18. Aguilera et al. [48]	USA Jan 2020 – Sep 2021	Retro SC	90, upto 360	≥ 18 AF abl	T 1,830 SDD 350 ONS 1,445	SDD 63.6 ± 9.6 ONS 66.4 ± 9.90		SDD 78.1 ONS 67.7		T 42.6 SDD 46.3 ONS 41.6	T 33 SDD 32.3 ONS 33.5	SDD 56.4 ± 8.75 ONS 55.23 ± 10.0	T 22 SDD 18.9 ONS 23.9		T 2.49 ± 1.54 SDD 2.03 ± 1.41 ONS 2.73 ± 1.48	NR	
19. Slingerland et al. [19]	Netherlands Jun 2018 – Dec 2020	Retro MC	3, 7, 120, 420	Index or re-do abl. NHR/DHD center (SDD) vs. national benchmark (ONS)	T 11,812 SDD 1,360 ONS10,452	Age ≥ 75 SDD 9.7% ONS 7.1%		SDD 68.6 ONS 66.2		NR	SDD 10.7 ONS 10.2	NR	NR		SDD ≥ 2: 53.4% ONS ≥ 2: 49.2%	NR	

d: Days; n: number; SD: Standard deviation; IQR: Interquartile Range; Parox: Paroxysmal; AF: Atrial fibrillation; HF: heart failure; LVEF: Left Ventricular Ejection Fraction; min: minutes; Prosp: Prospective; SC: Single-center; ADD: Anti arrhythmic drug; T: Total; SDD: Same-day discharge; ONS: Overnight stay; * Other data unavailable; UK: United Kingdom; MC: Multicenter; Sx: Symptomatic; y: year ; Excl: Excluded; Retro: retrospective; abl: ablation; LA: left atrial; USA: United States of America; AFL: Atrial flutter; Outpt: Outpatient; Match: Matched / paired cohort; BMI: Body mass index; AT: Atrial tachycardia; Parall: Parallel comparative cohort; LOS: Length of stay; CPT: Current procedural terminologyVCHR: Vancouver Coastal Health Region; w/: with; RF: Radiofrequency ablation; Cryo: Cryoballoon ablation; RCT: Randomized clinical trial; CrCl: creatinine clearance; NHR: Netherlands Heart Registration Center; DHD: Dutch Hospital Data Center

### Ablation procedures and same-day discharge protocols

Procedural characteristics and SDD protocols are summarized in Table 2. All studies evaluated ablation strategies centered on pulmonary vein isolation using radiofrequency, cryoballoon, or mixed-energy approaches, with additional lesion sets at operator discretion. Anesthetic strategies ranged from general anesthesia to conscious or deep sedation. Anticoagulation practices were generally consistent with contemporary standards, and vascular access was predominantly femoral, with hemostasis achieved through manual compression, figure-of-eight sutures, compression devices, or vascular closure systems.

SDD assignment varied across studies. Some programs used pre-procedural eligibility criteria and designated SDD as the default strategy for appropriate candidates, while others determined SDD post-procedure based on intraprocedural events, procedure end time, and early recovery. Most protocols required an observation period of 3 to 8 h with stable vital signs, no evidence of pericardial effusion or significant access-site bleeding, and successful ambulation without symptoms. Social and logistical requirements such as caregiver availability, reliable contact information, and proximity to the treating center, were frequently specified. Structured early follow-up, typically via telephone contact within the first days and at least one in-person visit within the first month, was common.

Across studies reporting SDD performance as a structured program, the pooled mean success rate was 89%. Failures of planned SDD were most often due to intraprocedural complications, concern for pericardial effusion or bleeding, persistent symptoms, anesthetic issues, late case completion, or limited social support.

**Table 2:** Technique and Same Day Discharge protocol

Study, year	Technique Energy,. Target. Anesthesia, Anticoag. Vascular ICE % % access, #						Timing of SDD assignment		SDD Criteria Elegibility Discharge			Monitoring Strategy	Success Rate, %	Failure Reasons	Length of stay in hours
1. Mondragon et al. [38]	RF/Cryo	PVI	GA	DOAC stop 24 h pre, restart 6 h post. UFH ACT 350–450 s	3 No US No VCD	Some		Inm PostPx		No intraPx compl	Stable 6 h	CCU Phonecall 1 wk Holter 30d	100	NR	SDD 10.9 ONS 25.03*
2. Opel et al. [39]	Cryo	PVI	CS	Unint (VKA or DOAC) 5000U UFH pre-TP + 4000U post-TP	1 No US Femostop	None		Inm PostPx		All	Stable 4 h, no PEff, groin OK, pt edu	4 h RU TTE PostPx 3 mo routine FU, ECG ± Holter.	98.6	NR	NR
3. Bartoletti et al. [40]	RF/Cryo	PVI ± AL	GA 46.2% CS 53.8%	Unint VKA (INR 2–3) NOAC (omit morning dose, resume evening) UFH ACT ≥ 300 s + protamine reversal	2–3 All US No VCD	None		PostPx		All morning cases (< 14:00 h) No mayor comorb	Stable 19:00–20:00 h, no compl, successful abl, no significant LV dysfn, no bleeding, pt edu	30 min RU 4 h bed rest ECG Nurse + EP review TTE if susp PEff 3mo clinic FU	100	NR	NR
4. Akula et al. [41]	RF	PVI ± AL CFAE	GA	UFH ACT 300–350 OAC resumed same day	5 No US No VCD	All		Inm PostPx		All No intraPx compl	Stable ≥6 h, no PEff on ICE, groin OK, pt edu	6 h CCU ECG Next day Phonecall 3mo FU	88	15 non-abl care 17 abl compl 4 pt preference 5 late cases	NR
5. Reddy et al. [42]	RF/Cryo	PVI ± AL CFAE	GA/CS	DOAC omit 1 dose VKA unint UFH ACT > 300 s + protamine reversal	NR Some US No VCD	None		PrePx		Soc support Op judge Frailty, comorb, travel dst, pt wishes	Stable 2 h bed rest + 1 h obs; groin OK; OAC restarted; caregiver present; pt edu	3 h ward; hourly vitals; ECG TTE if susp PEff 3–6 mo clinic	99	Late finish	NR
6. Deyell et al. [9]	RF 96.5 Cryo 3.5	PVI	GA 98.7	VKA 67.7%, Unint DOAC 23% interrupted, resumed 6 h postPx LMWH bridging 41.1%	2–3 Some US No VCD	Some		PostPx		All No intraPx compl	Stable ≤ 8 h, ambulation, no bleeding, pt edu	CCU, bedrest 3 h, recovery up to 8 h Phonecall 7-10d Clinic 3–6-12mo	79.2	22.8% access site 22.8% 23.9% late finish 10.1% intraPx compl 8% anesthesia 1.6% OSA monitor 5.2% arrhythmia 4.4% HF 3.8% Planned 19.5% other	NR
7. Creta et al. [11]	RF Cryo (SDD 79.2)	PVI ± subst	GA 8.4 CS 91.6	Unint VKA/DOAC UFH ACT 300–350 s, protamine at end	2–3 All US All Femostop or Z suture	Some		PostPx		Op discretion; soc/geo/clinical fcs (elder, comorb)	Stable vitals, ambul, groin OK, no bleeding, caregiver.	TTE in lab Ward bedrest 3 h, monitor 4–8 h. NEWS, ECG Clinic 3mo	NR	NR	NR
8. Kowalski et al. [10]	Cryo	PVI	GA	DOAC held night before, no bridging, continued ≥ 60 d UFH ACT ≥ 300 s	4 All US Some Fig. 8 suture	Some		Inm PostPx		No intraPx compl. Dist from hx	Stable vitals, ambul, no bleeding, pt edu, caregiver/transport arranged	End of px ICE or TTE CXR if PEff susp CCU 6 h 30 d Phone/clinic	NR	NR	NR
9. Rajendra et al. [43]	RF	PVI ± AL CTI	GA	Unint OAC UFH intraPx Colchicine 2 wk post-discharge	NR Some US All VCD (purse string)	NR		PrePx		Stable OAC (INR on target), no bleeding history, LV dysfn, pulm dis, or interventional px < 60d BMI < 35, CHA_2_DS_2_VASc ≤ 3 Proximity/logistics, Px before 1 pm	Stable, ambul, groin OK, PO, No intraPx compl, purse-string removed, pt edu, caregiver	6 h bedrest, 1–2 h ambul. Nextday & 7 d nurse call TTE 3 mo	93.2	1 pt preference 1 fever 1 groin bleeding	NR
10. He et al. [8]	RF/Cryo	PVI ± AL	GA 5 CS 95	VKA unint DOAC (held night before) UFH, protamine 50 mg reversal	NR NR US Some Fig. 8 suture	None		PostPx		Op discretion Logistics (dist/end time)	Stable vitals, ambul, no bleeding, caregiver, pt edu	CCU, bedrest 3 h, monitor ≤8 h TTE inm & 2–3 h Phonecall 7-10d Clinic 3, 6, 12 mo	79.2	34 compl 25 op discretion, 27 logistical (late finish/med delays)	NR
11. Brown et al. [45]	RF/Cryo	PVI 28.4%, PVI ± AL 54.5% Other: 17.1%	GA 32.5 CS 66.7	NR	NR NR US Some VCD	Some		PostPx		Per site protocols, crossovers without events excluded	Stable clinical exam, ambul, groin OK	2–4 h bedrest	70.8	NR	NR
12. Deyell et al. [15]	RF 80.5 Cryo 19.5	PVI	GA	VKA 6.9%, DOAC 89.1%. UHF, protamine reversal, OAC resumed 6 h postpx	NR Some US Some VCD	NR		PrePx		Default SDD; pre-planned ONS by Op discretion (complexity, soc support)	Stable vitals, ambul, no bleed, caregiver, pt edu	CCU, bedrest 3 h, recovery <8 h Phonecall 7-10d Clinic 3, 6, 12 mo	90.5	6 Access site 4 Anesthesia 11 intrpx compl 1 OSA monitor 1 arrhythmia 1 HF 1 respiratory 10 late finish 6 other	NR
13. Sangrigoli et al. [17]	Cryo RF for CTI	PVI ± AL CTI	GA	UFH ACT > 350 s, protamine reversal	NR NR Figure 8 suture, some VCD	NR		6 h PostPx		No intrPx compl, <4 h GA. LVEF > 40, CrCl > 50, CHA2DS2-VASc ≤ 3.	Stable, ambul, no bleed, SR, caregiver.	Recovery 6 h w/ambul at 3–4 h Clinic 2 wk, 1 mo	100	NR	NR
14. Eldadah et al. [36]	RF/Cryo	PVI	NR	OAC unint 83% Op discretion	3–4 NR US All VCD (VASCADE MVP)	NR		PostPx Post-ambul		Excl infx, PMH VTEV, bleeding diathesis, coagulopathy, hypercoagulability, platelets < 100,000, prev fem cath < 30d. Concomitant condition(s) that may delay ambul or discharge.	Stable vitals; ambul, no bleed, void, ECG OK, caregiver 24 h	Bedrest 2–2.5 h, observe 20 min post ambul ECG Phonecall 2-4d, 15 ± 5d	91.2	NR	NR
15. Asbeutah et al. [47]	RF 13 Cryo 87	NR	NR	NR	NR	NR		PostPx		All pandemic (minimum stay policy)	NR	NR	78.4	NR	NR
16. Rajendra et al. [44]	RF	PVI ± AL PW, CTI if FL	GA	Unint OAC UFH (ACT NR)	NR Some US VCD or purse string per Op	All		PrePx		Stable OAC, no bleeding, LVEF > 40%, no pulm dis, no px < 60 d, BMI < 35)	Stable; groin & respiratory ok, ambul, PO, purse-string removed, soc support, ambulation and oral intake; purse-string removed; adequate social support	ICE excl PEff Recovery unit 3–4 h. Phonecall next day (self exam) & 30d. Clinic 3, 6, 12 mo.	86.1	26.1% Late finish 70% soc support/distance 3.6% compl	NR
17. Castro-Urda et al. [18]	RF 8 Cryo 92	PVI	CS	Unint OAC (DOAC 84%, VKA 16%) UFH ACT > 300 s; protamine reversal in ONS.	2–3 Some US VCD Proglyde SDD Figure 8 ONS	NR		PrePx		All proglide arm. Excl: pregn, <1y expectancy, OAC contraindicate, GA, condition requiring admission	Stable, ambul, no bleed	CCU 3 h Phonecall 7, 30d	84	3 Proglide malfunction 3 Rhythm compl 1 groin bleed 1 susp PEff	SDD 5.89 ± 7.47 h ONS 27.09 ± 2.29 *p* < 0.005
18. Aguilera et al. [48]	RF 93.1 Cryo 7.7	PVI ± AL	GA	Unint OAC (AVK 10.5%)	NR All US Some VCD VASCADE MVP	All		PostPx		Px complexity, pt comfort, soc support (24 h caregiver, <2 h travel or hotel, op discretion. Excl of comorb	Stable, ambul, no bleed, PO, void, ECG ok, cargiver	Recovery unit Phonecall next day Rhythm transmitter 3mo	NR	NR	SDD 6.37 ± 1.24 ONS 29.61 ± 49.43 *p* < 0.001
19. Slingerland et al. [19]	RF: SDD 9.2 ONS 60.8 Cryo: SDD 90.8 ONS 39.2	PVI ± AL	GA (nurse monitored)	Unint OAC UFH ACT > 300 s; protamine reversal	2–4 No US No VCD, 6 h bandage	NR		PostPx		No intrPx compl Finish < 15:00 h	Stable, ambul, no bleed, bandage removed	TTE Ward, 6 h bandage removal, 1 h obs	NR	NR	SDD 0.50 days * ONS 1.52 days *

Anticoag: Anticoagulation; ICE: Intracardiac echo; ≠: Differences; RF: Radiofrequency ablation; Cryo: Cryoballoon ablation; GA: General anesthesia; PVI: Pulmonary vein isolation; PVI ± AL: Pulmonary vein isolation ± additional lesions; DOAC: Direct oral anticoagulant; UFH: Unfraction heparin; ACT: Activated clotting timg; US: Ultrasound; VCD: Vascular closure device; Inm: Inmediate; PostPx: Post-procedure; Compl: Complications; IntraPx: intraprocedural; h: hours; CCU: Cardiac Care Unit; * Other data unavailable; wk: weeks; d: days; NR: Not reported; CS: Controlled sedation; Unint: Uninterrupted; VKA: Vitamin K antagonist; PEff: Pericardial effusion; pt: Patient; edu: Education; RU: Recovery unit; mo: Months; ECG: Electrocardiogram; Comorb: Comorbidities; abl: ablation; LV: Left ventricular; dysfn: Dysfunction; min: Minutes; EP: Electrophysiologist; TTE: Trans-thoracic echocardiogram; susp: Suspected; CFAE: Complex fractionated atrial electrograms; OAC: Oral anticoagulation; PrePx: Pre-procedure; Soc: Social; Op: Operator; Dst: distance; Obs: Observation; Ambul: Ambulation; OSA: Obstructive sleep apnea; Subst: Substrate; Geo: Geographic; Fcs: Factors; NEWS: National Early Warning Score; Hx: Hospital; Px: Procedure; CXR: Chest X-Ray; CTI: Cavo-tricuspid isthmus; Pulm dis: Pulmonary disease; PO: Per os – tolerates oral intake; Med: Medication; SR: Sinus rhythm; Infx: Infection; PMH: Past medical history; VTEV: Venous thromboembolic events; Prev fem cath: Previous femoral catheterization; PW: Posterior wall; Others, per Table 1.

### Risk of bias

**Fig. 2:**
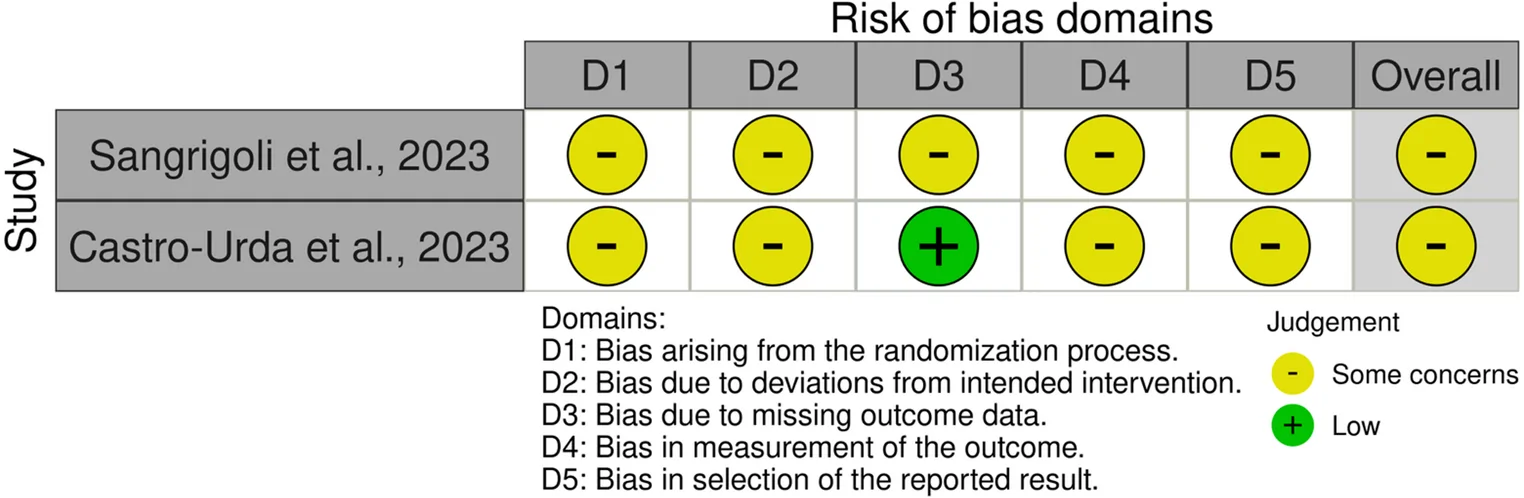
Risk of bias assessments for RCT (ROB2)

**Fig. 3:**
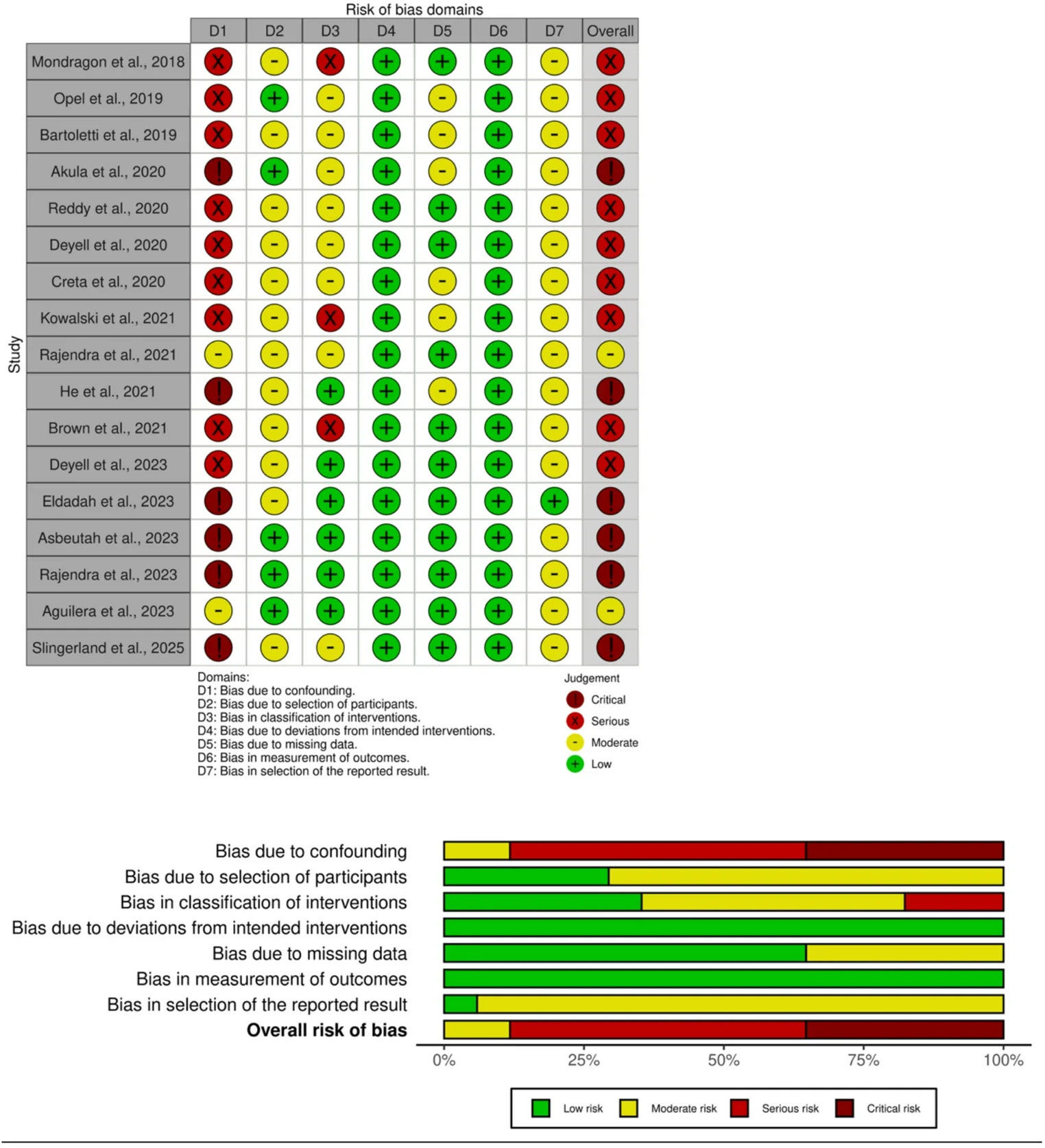
Risk of bias in non-randomized studies (ROBINS-I)

Risk-of-bias assessments are shown in Fig. 2. Both RCT were judged to have an overall risk of bias of “some concerns,” primarily related to the randomization process and lack of blinding. Risk of bias in non-randomized studies, assessed using ROBINS-I (Fig. 3), was predominantly moderate or serious, driven by systematic differences in the characteristics of patients assigned to SDD versus ONS, poor control of confounding variables, and heterogeneous assessment of post-procedural outcomes. Six studies were judged to have critical overall risk of bias.

### Critical risk of bias explanations

Akula et al. [41]: Critical risk due to historical confounding: the before-and-after design mixes changes in operator experience, technique, and anticoagulation with the introduction of SDD, making attribution impossible.

He et al. [8] Critical risk because the decision for SDD was entirely physician-driven without predefined criteria or adjustment, resulting in extreme confounding and unbalanced groups.

Eldadah et al. [46] Critical risk since patient selection and exclusion of early adverse events made the SDD cohort inherently lower-risk, invalidating direct safety comparison.

Asbeutah et al. [47] Critical risk from severe selection bias and unadjusted baseline differences between SDD and ONS groups, with key confounders uncontrolled.

Rajendra et al. [44] Critical risk because exposure classification depended on post-procedure clinical stability, excluding complicated cases and biasing results toward SDD safety.

Slingerland et al. [19] Critical risk owing to protocol-driven exclusion of early complications and differential follow-up, which systematically favors the SDD group.

### All-cause mortality

**Fig. 4:**
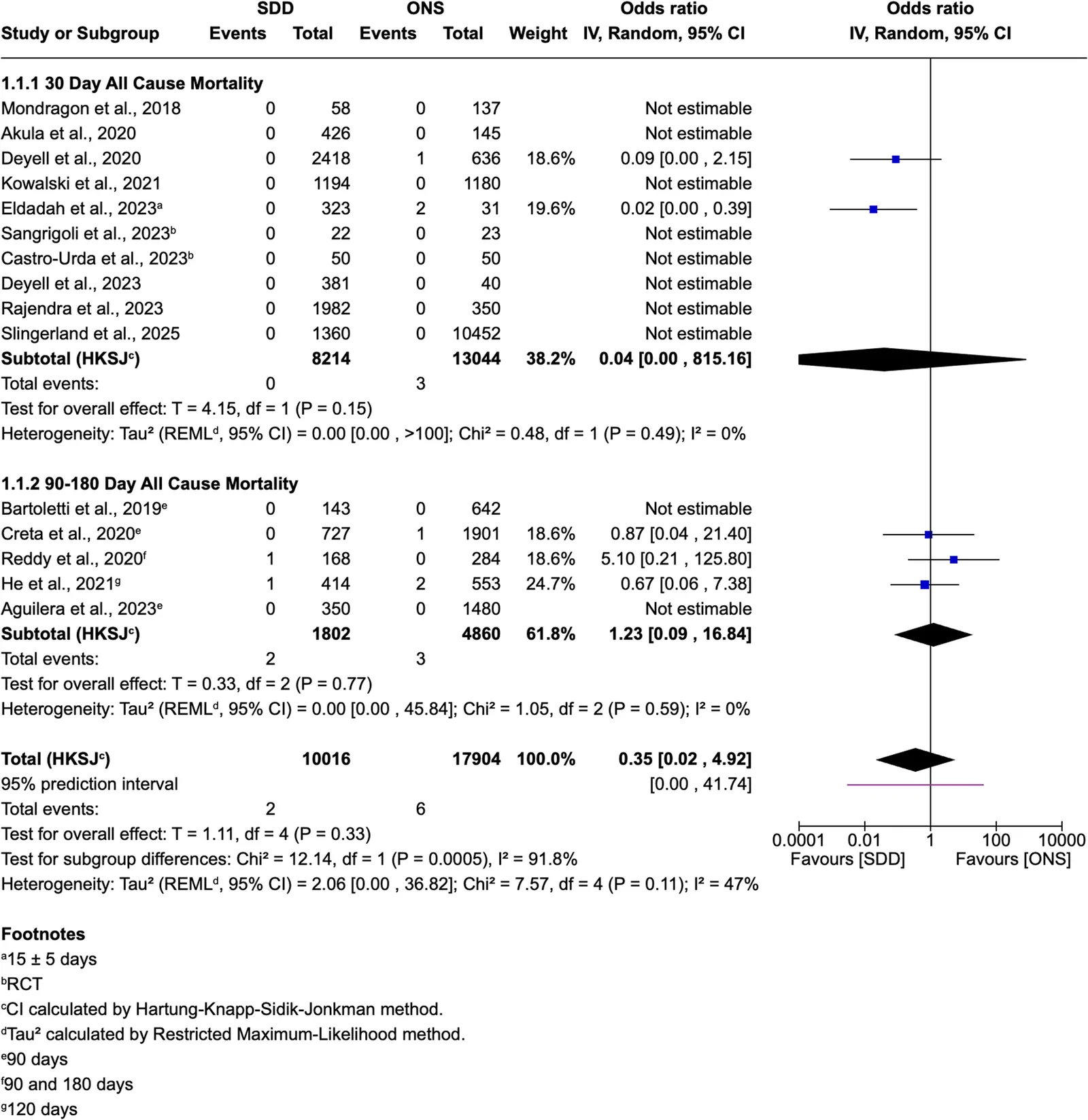
All-cause mortality forest plot

All-cause mortality was infrequently reported, and events were rare in both groups. Several studies reported zero deaths in one or both groups. Comparisons with zero events in both arms were not included in pooled estimates but were retained in descriptive summaries, which contributed to imprecision. In the random-effects meta-analysis, the pooled odds ratio did not differ significantly between SDD and ONS (OR 0.35; 95% CI 0.02–4.92; I^2^ 47%), although precision was limited by very low event rates (Fig. 4). Subgroup analyses by follow-up duration did not reveal consistent differences between SDD and ONS (Fig. 4).

### Unplanned medical contact

**Fig. 5:**
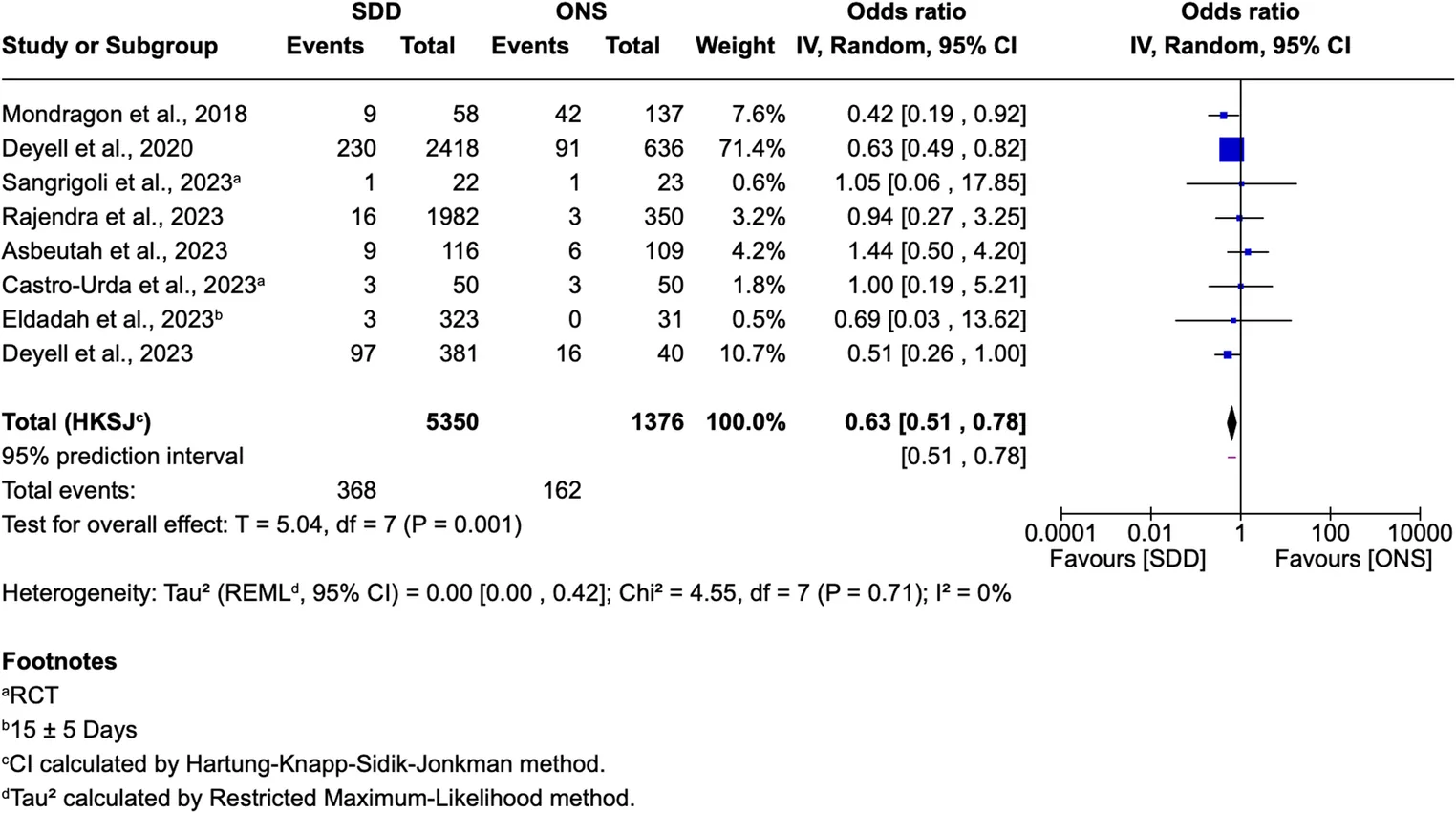
30-day only Unplanned Medical Contact

Unplanned readmissions and ED visits were the most consistently reported outcome. In the random-effects model restricted to short-term (30-day) follow-up, SDD was not associated with higher odds of unplanned healthcare use and the pooled estimate favored SDD (OR 0.63; 95% CI 0.51–0.78; I^2^ 0%) (Fig. 5). Most contributing studies reported crude event counts, and adjusted estimates were used when available. In exploratory analysis including different time horizons, pooled estimate consistently favored SDD (OR 0.77; 95% CI 0.65–0.92; I^2^ 23%) and earlier (30-day) follow-up windows showed more pronounced effects favoring SDD, with attenuation over longer (90–120 day) temporal horizons (OR 0.93; 95% CI 0.57–1.52]; I^2^ 25%) (Supplementary Fig. 1). Sensitivity analyses restricted to randomized trials (Supplementary Fig. 2) or to studies using protocolized pre-procedural SDD assignment (Supplementary Fig. 3) produced consistent but less precise estimates. Subgroup analysis according to routine pre-discharge transthoracic echocardiography, showed similar point estimates, with overlapping confidence intervals and no clear evidence of effect modification (Supplementary Fig. 4). Excluding studies at critical risk of bias, the results were again consistent without changing the direction of effect (Supplementary Fig. 5).

### Procedural complications

Procedural and post-discharge complications are summarized in Table 3. Definitions, severity thresholds, and follow-up windows varied substantially across studies, precluding quantitative synthesis. Overall complication rates were low in both groups, although four studies showed higher event rates in the ONS arm. In Akula et al. [41] and Kowalski et al. [10], overnight admission reflected clinical concern and concentrated older, higher-risk patients. Eldadah et al. [46] applied broad composite outcomes and subacute follow-up windows, increasing event counts compared with other cohorts. Rajendra et al. [44] used strict SDD eligibility criteria that selected lower-risk patients, leaving a higher-risk ONS group. These patterns suggest that the excess events in ONS reflected case-mix and outcome definition differences rather than an adverse effect of SDD. Major complications, including tamponade, stroke, and significant vascular injury, remained uncommon across studies.

**Table 3:** Complications

Study, year	Follow Up Time, days	Same-Day Discharge, *n*/total (%)	Overnight Stay, *n*/total (%)	*p* value**	Definition
Mondragon et al. [38]	30	2/58 (3.4)	7/137 (5.1)	0.900	Vascular and pericardial
Opel et al. [39]	90	15/276 (5.4)	17/276 (6.2)	NSS*	Phrenic nerve palsy, pericarditis, pericardial tamponade or effusion, vascular complication, bradycardia, air embolism
Bartoletti et al. [40]	90	5/143 (3.5)	3/642 (0.5)	0.430	Minor (Vasovagal events, minor femoral bleeding, sore throat, nausea/vomit, pericarditic chest pain)
Akula et al. [41]	30	23/426 (5.4)	17/145 (11.7)	**0.020**	Major (bleeding requiring transfusion, pericardial effusion & drainage, transient ischemic attack) or Minor (hematoma, others not specified)
Reddy et al. [42]	90–180	4/168 (2.4)	11/284 (3.9)	0.680	Phrenic nerve palsy, migraine, atrio-esophageal fistula and death, pulmonary oedema, minor hematoma, arteriovenous fistula requiring surgery, pseudoaneurysm treated with thrombin injection, tamponade, transient ischemic attack, air embolism, temporary numbness.
Deyell et al. [9]	30	9/2418 (0.4)	4/636 (0.6)	0.990	Composite safety outcome (death, stroke/transient ischemic attack or embolism, and bleeding requiring hospitalization)
Kowalski et al. [10]	30	15/1194 (1.3)	24/1180 (2.0)	**0.005**	Hematoma, phrenic nerve palsy, transient ischemic attack, chronic heart failure, pericardial effusion, pericarditis, death, others.
Rajendra et al. [43]	90	2/41 (4.9)	2/41 (4.9)	NR	Hematoma, gastrointestinal bleeding, AV block.
He et al. [8]	1	0/414 (0) 22/414 (5.3)	3/553 (0.5) 9/553 (1.6)	0.265 **0.001**	Major (vascular injury requiring surgery, bleeding requiring transfusion) Minor (Bleeding, hematoma, pericardial effusion, reversible AV node injury)
Brown et al. [45]	3	30/210 (14.3)	25/199 (12.6)	0.665	Major (stroke, tamponade, phrenic nerve palsy, sinus node dysfunction, esophageal perforation) or Minor (unstable vitals, access site issues, pericarditis, simple effusion, unstable labs or vitals, incomplete studies, acute kidney injury and others)
Deyell et al. [15]	30	4/381 (1.0)	0/40 (0)	0.324	Composite safety outcome (death, stroke/transient ischemic attack or embolism, and bleeding requiring hospitalization)
Sangrigoli et al. [17]	30	0/22 (0)	0/23 (0)	NR	Serious adverse outcomes (complications requiring medical-surgical intervention, inpatient stay or prolonged hospitalization [> 24 h], permanent injury or impairment, or death)
Eldadah et al. [46]	15 ± 5	9/323 (2.8)	16/31 (51.6)	**< 0.001**	Adverse events (Bleeding, hematoma, arteriovenous fistula, nerve damage, thrombosis, arrhythmia, urinary tract infection, hypotension, congestive heart failure, hemodynamic instability, aspirate pneumonia, stroke, syncope, acute kidney injury.
Rajendra et al. [44]	30	30/1982 (1.5)	14/350 (4.0)	0.452	Subacute (Vascular access complications [hematoma, pseudoaneurysm and arteriovenous fistula], bleeding, pericardial effusion/tamponade, pericarditis, acute HF exacerbation, stroke within 7 days, atrioesophageal fistula, phrenic nerve injury, pulmonary vein stenosis, pneumothorax, atelectasis, pneumonia, and death.
Castro-Urda et al. [18]	30	7/50 (14)	3/50 (6.0)	0.182	Acute (temporary phrenic nerve paralysis, sinus pause, nodal rhythm, ventricular tachycardia, tamponade suspicion that required pericardial access)
Aguilera et al. [48]	30	7/350 (2.0)	53/1480 (3.6)	NR	Adverse events (Volume overload, anesthetic complication, arrhythmia, bleeding, pericarditis, urinary tract infection without sepsis.
	90	2/350 (0.6)	38/1480 (2.6)	NR	Major (cardiogenic shock, hemothorax, pericardial effusion requiring evacuation, phrenic nerve injury, pseudoaneurysm, stroke or transient ischemic attack, urinary tract infection with sepsis, vascular injury requiring intervention.)
19. Slingerland et al. [19]	3	2/1360 (0.1)	17/10,452 (0.2)	0.893	Thromboembolic
	30	14/1360 (1.2) 5/1360 (0.4)	119/10,452 (1.5) 48/10,452 (0.6)	0.720 0.634	Vascular Tamponade

NSS, not statistically significant; NR, not reported. *p value reported for each type of complication, none was statistically significant. ** p values as reported by the authors in each study.

## Discussion

In this systematic review and meta-analysis of 19 studies including 26,335 patients, protocolized same-day discharge after atrial fibrillation ablation was not associated with higher all-cause mortality, short-term unplanned medical contact, or major complications compared with overnight stay. Mortality events were rare, contributing to imprecision. For unplanned healthcare utilization at 30 dyas, the pooled odds ratio favored same day discharge without evidence of excess events, though most nonrandomized studies carried moderate to serious risk of bias. Overall certainty remains limited by the predominance of nonrandomized evidence and residual confounding. Nevertheless, in experienced centers using structured pathways and careful patient selection, the available evidence supports same-day discharge as a reasonable default strategy for appropriate outpatients, with overnight observation reserved for higher-risk profiles, intraprocedural concerns, or logistical barriers to safe discharge.

Three systematic reviews published in 2022 also reported no significant differences in short-term complications or readmissions, but all relied on retrospective cohorts in which SDD was defined only by observed discharge timing. This design introduces structural selection bias and exposure misclassification, limiting their ability to evaluate SDD as a reproducible clinical strategy [12–14]. The recent review performed by the European Heart Rhythm Association Health Economics Committee reached similar conclusions, reporting low rates of post discharge complications, rehospitalizations, emergency visits, and mortality with same day discharge and emphasizing the need for randomized evidence [49]. It was published after registration of our protocol and outside our initial search window, but it underscored the need for an updated synthesis incorporating the prospective cohorts and randomized trials published thereafter. They included studies lacking true concurrent control groups and others in which SDD was classified solely by observed discharge time rather than a predefined protocol, introducing substantial indication bias. Several cohorts were derived from administrative or registry datasets with limited clinical adjudication, increasing misclassification risk and reducing internal validity.

Our review extends the evidence base by incorporating two randomized trials, several recent prospective cohorts, and a wider range of contemporary protocols, while restricting inclusion to studies that described a reproducible same day discharge pathway with explicit eligibility and workflow criteria and used clinically adjudicated outcomes. This approach focuses on same day discharge as a defined intervention rather than a post hoc discharge label.

Large real-world analyses and economic evaluations provide complementary context but were excluded from quantitative synthesis because of design limitations. The national registry study by Sandhu and colleagues used the NCDR atrial fibrillation ablation registry to show substantial growth in same day discharge across the United States and very low major complication rates yet relied on administrative coding to define exposure and outcomes and lacked information on structured protocols [50]. The single center propensity adjusted study by Zenger and colleagues reported noninferior adverse events and similar total procedural costs between same day discharge and overnight observation, with a trend toward better patient reported outcomes in the same day group, but discharge decisions were operator-selected rather than protocol-driven, limiting causal interpretation [51]. These data, together with contemporary prospective cohorts that describe high rates of successful same day discharge and high patient satisfaction under standardized protocols, support the overall safety signal observed in our analysis while also underscoring the importance of programmatic implementation.

Our findings have several implications for clinical practice and service planning. First, they align with consensus recommendations from the HRS and ACC scientific statement, which advocates structured checklists that confirm hemostasis, hemodynamic stability, pain control, absence of pericardial effusion, adequate social support, and timely access to urgent care before same day discharge after intracardiac ablation [52]. The approximately 89% success rate across structured programs shows that SDD can be reliably implemented in centers that apply standardized criteria and early follow-up. Second, our data suggest that same day discharge, when implemented as a pathway that includes early follow up and clear instructions for patients, does not increase unplanned healthcare utilization and may reduce emergency visits and short readmissions, consistent with reports of stable or modestly reduced costs.

This review has key methodological strengths. We used a prospectively registered protocol, comprehensive multi database search and risk of bias assessment with contemporary tools including RoB 2 and the updated ROBINS I V2 framework [23–25, 33–35]. We applied strict eligibility criteria that excluded claims only datasets and studies that classified same day discharge solely on observed discharge time without a structured protocol, in order to reduce exposure misclassification and indication bias and to better approximate the effect of a reproducible clinical strategy. Our synthesis separated randomized from nonrandomized evidence, reported prediction intervals alongside pooled estimates, and summarized key protocol components.

Several limitations remain. Most included studies were nonrandomized and at moderate or serious risk of bias from confounding and selection, since patients chosen for same day discharge tended to have fewer comorbidities, shorter procedures, and fewer intraprocedural issues than those admitted overnight, even when multivariable adjustment or propensity methods were used. The two randomized trials were small and had some concerns regarding randomization, blinding, and adherence to assigned strategies, which limits confidence in the experimental evidence [17, 18]. Outcome definitions, follow up windows, and composite endpoints varied across studies, precluding pooled analysis of complications and contributing to heterogeneity. Several prespecified subgroup analyses could not be performed because studies did not report outcomes separately by AF type, monitoring duration, or hemostasis strategy, and only a few studies used a single ablation energy source while also reporting the outcomes of interest. As a result, we could not determine whether the effect of SDD differs by paroxysmal versus persistent AF, shorter versus longer post-procedural observation, or specific vascular-closure approaches. None of the included studies evaluated pulsed-field ablation, which limits applicability to contemporary workflows incorporating this modality. Our analyses relied on study level data, which restricted exploration of effect modification by factors such as heart failure status, frailty, or social vulnerability. Finally, most protocols excluded high risk patients, inpatients, and those with significant logistical constraints, so our conclusions apply primarily to selected outpatients treated in experienced centers and should not be extrapolated to all patients undergoing atrial fibrillation ablation. Accordingly, these findings should not be generalized to patients with advanced heart failure, substantial frailty, complex or prolonged ablation procedures, or limited access to urgent reassessment after discharge, for whom overnight observation may remain clinically appropriate.

Taken together, the available evidence suggests that structured same day discharge protocols for atrial fibrillation ablation, when applied to appropriately selected patients in experienced centers, achieve high rates of successful discharge without a signal of excess mortality, serious complications, or short-term unplanned healthcare utilization compared with overnight stay. Although certainty is constrained by observational designs, confounding bias, and heterogeneity in pathways and outcome definitions, the consistency of findings across contemporary cohorts and randomized data supports implementation of same-day discharge as the default for eligible patients within standardized programs. Larger pragmatic randomized trials and high-quality registries incorporating patient reported outcomes, protocol details, and economic endpoints are needed to refine patient selection, evaluate safety in higher risk groups and emerging technologies, and guide future recommendations.

## Conclusion

Same day discharge after atrial fibrillation ablation, when delivered through structured protocols and applied to selected outpatients, has reproducible success rates and does not show an excess of mortality, major complications, or short-term unplanned healthcare contact compared with overnight observation. Precision for uncommon outcomes remains limited, and most available evidence is derived from observational cohorts with residual selection bias. In this context, same-day discharge can be considered the default approach for appropriate patients in experienced centers that use explicit eligibility criteria, standardized post-procedural assessment, and early follow-up, while overnight observation remains appropriate for higher-risk clinical profiles, intraprocedural concerns, or logistical constraints. Additional randomized trials and robust prospective registries are needed to strengthen certainty, assess safety in higher-risk populations as well as newer ablation modalities, and define implementation standards across settings.

### Capsule summary

Structured same-day discharge after atrial fibrillation ablation is feasible and appears safe in eligible outpatients, with no signal of increased mortality, major complications, or short-term unplanned medical contact compared with overnight observation. In experienced centers using standardized criteria and early follow-up, same-day discharge can be implemented as the default strategy, with overnight stay reserved for higher-risk patients or procedural and logistical concerns.

## Supplementary Information

Below is the link to the electronic supplementary material.

Supplementary Material 1

## Data Availability

Full study protocol as well as all statistical analysis will be available at request.
