# Supplementary material for: Same-day discharge versus overnight stay after catheter ablation for atrial fibrillation: a systematic review and meta-analysis
Source: J Interv Card Electrophysiol. 2026 May 18;69(8):2607–23. doi: 10.1007/s10840-026-02286-x (PMC13739960; doi:10.1007/s10840-026-02286-x)
Supplement: Supplementary file 1 [file 10840_2026_2286_MOESM1_ESM.docx]

Supplementary Figure 1. Readmissions and emergency department visits forest plot, all time horizons

Supplementary Figure 2. Readmissions and emergency department visits forest plot – RCT only

Supplementary Figure 3. Readmissions and emergency department visits forest plot – Protocolized pre-procedural SDD assignment

Supplementary Figure 4. Readmissions and emergency department visits forest plot – Routine pre-discharge transthoracic echocardiography

Supplementary Figure 5. Readmissions and emergency department visits forest plot – Exclusion of critical risk of bias
